# Osteoporotic vertebral fractures: an update

**DOI:** 10.1051/sicotj/2025035

**Published:** 2025-07-16

**Authors:** Ioannis I. Daskalakis, Johannes D. Bastian, Andreas F. Mavrogenis, Theodoros H. Tosounidis

**Affiliations:** 1 Department of Orthopaedic Surgery, Medical School, University of Crete 71110 Heraklion Greece; 2 Department of Orthopaedic Surgery and Traumatology, Inselspital, Bern University Hospital, University of Bern 3010 Bern Switzerland; 3 First Department of Orthopaedics, National and Kapodistrian University of Athens, School of Medicine 15562 Athens Greece

**Keywords:** Osteoporosis, Spine, Vertebral fractures, Fracture management, Secondary fracture prevention

## Abstract

*Introduction*: Osteoporotic vertebral fractures (OVFs) are the most common type of fragility fractures. They have a significant and usually detrimental impact on the patient’s functional status and mortality rate, constituting a substantial burden for the patients, their families, and the healthcare system. This narrative review aims to summarize the current knowledge of osteoporotic vertebral fractures and secondary fracture prevention. *Methods*: A comprehensive literature search was conducted across major medical databases, including PubMed, Scopus, and Web of Science. Relevant studies, guidelines, and reviews published were analyzed to provide a broad perspective on the topic. *Results*: Diagnosis of OVFs is based on history, clinical examination, and plain lateral radiographs of the spine. Their management is mainly non-operative, with surgery being reserved for specific indications. Successful management of osteoporotic vertebral fractures entails alleviating pain, early restoration of mobility, and secondary fracture prevention. Prevention of the next osteoporotic fracture is paramount and should be an integral element of their management. The Fracture Liaison Service (FLS) is the main contemporary service that serves this purpose. *Discussion*: Diagnosis of OVFs is simple but requires vigilance from the clinicians. Early, accurate diagnosis is essential to initiate appropriate treatment and provide the opportunity for secondary fracture prevention.

## Introduction

Major osteoporotic fractures are fractures of the hip, spine, wrist, and humerus [1]. Osteoporotic vertebral fractures (OVFs) are the most common type of single osteoporotic fracture, with 1.5 million cases being reported annually worldwide [2]. An OVF is diagnosed when a radiographic abnormality is present after a minor injury [2]. They affect the anterior column of the vertebra, leaving the posterior elements of the spine intact. In osteoporotic patients, minor injuries resulting in fractures can occur with daily activities, such as lifting objects, bending forward, or sitting on a low chair [2]. OVFs are a major source of disability, as they can cause persistent back pain and kyphotic deformities that have a significant impact on the patients’ functional status and mortality rate [3, 4]. Recent evidence suggests that they frequently remained undiagnosed [5]. The consequences of underdiagnosis are severe, as patients do not receive appropriate treatment and are not referred for osteoporosis screening and treatment initiation. Consequently, the secondary fracture prevention opportunity is lost, and patients are readmitted to the hospital with major secondary fractures [6]. This review aims to summarize the current concepts of osteoporotic vertebral fracture management and secondary fracture prevention.

## Epidemiology and economic burden

The true prevalence/incidence of OVFs is probably underestimated, as in one-third of the cases, there are no significant clinical symptoms, and less than 10% of the patients are admitted to the hospital [7]. The reporting of OVFs varies depending on whether the definition of a fracture is clinical or radiological [7]. Prevalence rates are similar worldwide, with the highest-to-lowest ratio between countries, within and across continents, varying from 1.4 to 2.6 [8]. The thoracolumbar junction (T12 to T2) is affected in 60–75% of the cases, followed by the L2 to L5 region in 30% [2].

Inevitably, OVFs pose a significant economic burden to healthcare systems worldwide. It has been estimated that the cost of all fragility fractures for healthcare systems within the EU was € 56.9 billion in 2019, with vertebral fractures accounting for 16% of all fragility fractures during that year [9, 10].

## Classification

Numerous OVF classification systems based on imaging features have been developed over the last decades [11–15]. Nevertheless, only a few of them have gained international acceptance and remain a subject of debate among experts [16].

Quantitative, qualitative, and combined methods with respective advantages and limitations have been used in the existing classification systems [16]. A summary of OVFs’ classifications is provided in Table 1.

**Table 1:** Summary of osteoporotic vertebral fractures classification systems.

Method	Characteristics	Examples	Description	Advantages	Disadvantages	Use
Quantitative	Measurement of height or surface without assessment of vertebral morphology	Eastell et al. [11]	Eastell et al.: three types of fractures depending on the decreased vertebral height (wedge, biconcave, crush fracture)	Objective, reproducible, use in automatized algorithms	High number of false positives and false negatives	Automated diagnosis, research
		McCloskey et al. [12]	McCloskey et al.: further classification of the wedge type into anterior and posterior			
Qualitative	Step-by-step analysis focused on vertebral abnormalities; exclusion of abnormalities caused by conditions other than OVF	Sugita et al. [13]	Classification of OVFs into five types according to morphology: type I (swelled-front), type II (bow-shaped), type III (projecting), type IV (concave), type V (indented)	Diagnosis of OVF without significant loss of vertebral height	Need for trained observer	Daily clinical practice
Combined	Description of the vertebral morphology combined with quantification of height or surface loss	Genant et al [14]	Genant et al.: determination of vertebral height or surface loss (degrees 0–3), 3 types of morphological deformity (wedge, biconcave, crush)	Simplicity, high inter- and intra-observer agreement	Confusion with anatomical variants and other conditions, need for trained observers	Daily clinical practice, research
		DGOU classification [15]	DGOU: Classification of OVFs into five types, according to CT and MRI findings			

## Diagnosis

The main complaint of a patient with an OVF is back pain, even though two-thirds of these fractures are asymptomatic [7]. A detailed history and clinical examination are essential to confirm that the cause of pain is indeed the fracture. Physical examination is often unremarkable, but it may reveal midline spine tenderness or kyphosis. In older adults, a significant limitation of mobility caused by pain may lead to shortness of breath or even decubitus ulcers. In approximately 2% of the cases, patients may present with neurologic deficits due to spinal cord compression [17]. The initiation of symptomatology is often insidious and may occur several months post-fracture. Symptoms include loss of sensation, urinary retention, sphincter dysfunction, and hyporeflexia or hyperreflexia [17].

OVFs are easily detectable in lateral radiographic views of the spine. A compression fracture is defined as a decrease in anterior, middle, or posterior height of at least 20%, or a decrease of at least 4 mm compared with baseline height (estimated based on vertebral body heightcan also distinguish benign from malignant fractures, estimate the age of the fracture of upper or lower vertebra). The osseous anatomy of the fracture can be better depicted with a CT scan. However, the fracture age cannot be reliably estimated, and the patient is exposed to a significant amount of radiation [18].

In fractures with less marked collapse, magnetic resonance imaging (MRI) can assist in confirming the diagnosis. MRI can also distinguish benign from malignant fractures, estimate the age of the fracture, and assess spinal stability, which is crucial for decision-making [19]. Finally, MRI can provide information about possible spinal cord and nerve root involvement [19]. OVFs can also be diagnosed with bone scintigraphy when MRI is contraindicted [20]. Scintigraphy can also differentiate malignant and benign fractures [20]. Nevertheless, when more than two vertebral bodies are involved, the possibility of confirming the hot uptake lesion as a new fracture by scintigraphy is low. Therefore, an MRI is then required [20].

## Management

The goal of OVF treatment is to minimize pain, restore mobility, and prevent the second subsequent fractures [21]. The majority of patients respond well to non-operative management, and surgery is reserved for patients with persistent pain despite conservative treatment and when there is a concomitant neurologic deficit [22].

### Non-operative management

Non-operative management is indicated as the first-line treatment for OVFs [23]. It consists of analgesia, the application of spine orthoses, and physical therapy [23]. Appropriate pain control is the first essential step, as it facilitates early mobilization and participation in physical therapy. Analgesia should be gradually tapered, provided that pain improvement is evident. In the majority of cases, a significant decrease in pain is expected within the first 4 weeks [24].

The goal of orthoses used in OVFs is to limit the range of motion, improve posture, minimize secondary traumatic injury, and alleviate pain. Bracing is recommended for the first 6–8 weeks after the injury until acute pain is resolved [25]. A recent systematic review demonstrated that the use of braces is beneficial in older adults, as it offers biomechanical vertebral stability, reduces kyphotic deformity, enhances postural stability, and muscle strength, and is associated with superior functional outcomes [26].

To achieve high patient compliance, the brace should be light and easy to put on, and should not compromise respiratory function. For thoracic fractures, thoracolumbar orthoses are usually utilized. Three-point hyperextension braces, such as the Jewett and the cruciform anterior spinal hyperextension (CASH) brace, are the most commonly used as they provide adequate hyperextension of the spine and stabilization of the injured segment [27]. Lumbosacral orthoses are available for lumbar fractures, but they can only immobilize the sagittal plane in the upper lumbar spine (L1–L3) while increasing the intervertebral motion of the L4–S1 levels [28].

Early mobilization within the limits of pain should be initiated as soon as possible. Initially, patients should be educated on how to avoid pain during daily activities [27]. Various exercise regimens, such as spinal extensor strengthening [29] and dynamic proprioception training, have been proposed [30]. However, a recent review reported that there is not sufficient evidence related to the benefits of exercise in patients with vertebral compression fractures [31]. Further research with high-quality randomized trials is necessary to determine the effectiveness of exercise [31].

### Operative management

The three main indications of operative treatment are painful OVF refractory to medical management, vertebral bodies weakened by neoplastic lesions, and symptomatic vertebral body microfracture [32]. Failure of non-operative treatment is defined as the inability to ambulate due to pain from a weakened/fractured vertebral body after 24–48 h of potent analgesic therapy, significant pain from a weakened/fractured vertebral body with no response to bed rest, bracing, analgesia and physical therapy, and unacceptable side effects due to the analgesic therapy necessary to reduce pain to a tolerable level [32].

Several risk factors associated with the failure of non-operative treatment have been identified, both patient and fracture-related [15, 33]. Clinical and radiographic factors along with a proposed risk stratification model to guide treatment selection are summarized in Table 2.

**Table 2:** Summary of clinical and radiographic risk factors and risk stratification.

Risk category	Clinical factors	Radiological factors	Recommended management
Low-risk	Pain adequately controlled with oral analgesics No neurological deficits Ability to ambulate independently Low burden of comorbidites Osteopenia (T-score > −2.5) Absence of frailty indicators	Vertebral body height loss <25% Stable fracture morphology (absence of posterior wall involvement or retropulsion)	Non-operative
Intermediate risk	Persistent or progressive pain beyond 4–6 weeks despite conservative measures Reduced ambulation ability and performance of daily tasks Early signs of frailty (sarcopenia, recent falls) Mild-to-moderate burden of comorbidities Osteoporosis (T-score −2.5 to −3.5) Absence of neurologic deficits	Vertebral body height loss between 25% and 50% Emerging or progressive segmental kyphosis	Initial course of non-operative management. Consideration of vertebral augmentation in case of persistent pain or radiographic progression of deformity
High risk	Escalating pain, refractory to medical therapy Neurological involvement Significant frailty or dependency in daily activities High comorbidity burden Severe osteoporosis density (T-score ≤ −3.5) Documented failure of non-operative management	Vertebral body collapse greater than 50% Radiographic evidence of posterior wall disruption or canal compromise	Operative management with vertebral augmentation or decompression and instrumentation, according to the neurologic status

The mainstay of operative treatment of OVFs is the vertebral augmentation techniques, which aim to consolidate the fracture and restore vertebral height. These techniques are percutaneous vertebroplasty and percutaneous balloon kyphoplasty [33]. The mechanism of pain relief provided is a combination of improved spine biomechanics after the cement injection, the chemical toxicity, and the exothermic effect of cement polymerization on nerve endings. Spinal instrumentation is indicated in patients with unstable fractures, chronic vertebral pseudarthrosis, and neurologic deficits [34].

Percutaneous vertebroplasty is the injection of radiolucent cement into the affected vertebra under imaging guidance [33]. It is indicated for the treatment of painful acute and subacute OVFs in patients who have failed to respond to a 4-to-6-week course of appropriate medical therapy. Vertebral fractures due to metastatic disease, multiple myeloma, and aggressive painful hemangiomas can also be treated with vertebroplasty [35, 36]. Absolute contraindications of this technique include asymptomatic vertebral fractures and the absence of bone marrow edema in the targeted vertebral body on MRI short tau inversion recovery (STIR) sequence images. The treatment of asymptomatic OVFs is also not an acceptable indication. Additional absolute contraindications include uncorrectable coagulopathy, active local or systemic infection and allergy to bone cement products. Relative contraindications are disruption of the posterior vertebral body wall, tumor extension into the spinal canal and very severely compressed vertebral fractures, defined as vertebral body collapse to less than one-third of the original height [37]. The optimal timing of vertebroplasty remains controversial, but it is generally accepted that it should be performed within four months after the fracture [38]. In fractures older than 4 months, percutaneous vertebroplasty could be performed only if there is persistent edema on MRI or bone scintigraphy [39].

Percutaneous balloon kyphoplasty is a vertebral augmentation technique that involves inflating a balloon inside the affected vertebra before cement injection [40]. The indications of kyphoplasty are the same as for vertebroplasty [40]. Kyphoplasty can additionally be performed in fractures with retropulsed fragments, as the elevation of the depressed endplates can result in the reduction of the fracture. The optimal indication of kyphoplasty is acute traumatic fractures (7–10 days) to better restore the vertebral height [33].

Several bone cement types are commercially available and vary in cost, radio opacity, rate of polymerization, and biocompatibility, with their advantages and disadvantages extensively described [41]. Despite the advent of newer composite and calcium phosphate cement, polymethyl methacrylate (PMMA) remains the most widely used in the treatment of osteoporotic and malignancy-related vertebral body fractures. It has been suggested that low cement volumes may be associated with worse clinical outcomes, but the ideal cement volume for vertebroplasty is still controversial [42].

The outcomes of vertebral augmentation compared to non-operative treatment have been a topic of significant controversy. Over the last decade, multiple randomized controlled trials (RCTs) have been conducted [43]. Published reviews included only one treatment arm or only one outcome variable. A recent systematic review by Halvachizadeh et al. included all treatment arms and three significant outcome variables: long-term pain, adjacent level fracture risk, and quality of life [43]. The study revealed that operative treatment resulted in greater improvement in pain and overall function. There were no significant differences in adjacent level fractures and quality of life measured with various tools [43]. Additionally, a meta-analysis by Hinde et al. that included more than two million vertebral fracture patients demonstrated that vertebral augmentation provided a 22% reduction in mortality compared to non-operative treatment at up to 10 years of follow-up [44].

Kyphoplasty and vertebroplasty have been compared in multiple studies. The effects of kyphoplasty and vertebroplasty have been compared in three RCTs [45–47]. Their characteristics are summarized in Table 3. Only the study of Dohm et al. favored kyphoplasty as it was associated with fewer cement leakage events, longer fracture-free survival, and less postsurgical loss of kyphotic-deformity correction during 2 years [46]. No other significant differences were detected in the outcome measures. The overall complication rates are low for both procedures, with complications occurring in less than 1% of the cases [48]. The most common complications are cement leakage, infection, and reactions to anesthesia [48]. More serious complications are rare and include neurologic deficits resulting from nerve injury, fractures of the rib, sternum or pedicle, pulmonary embolus, hemothorax, pneumothorax, or cement embolism [48]. Although asymptomatic leakage of cement has been reported at higher rates in vertebroplasty (34% vs. 9%), the symptomatic complication rates are similar between the two procedures [49].

**Table 3:** Summary of randomized studies comparing vertebroplasty to kyphoplasty.

Author	Year	Country	Total number of subjects	Vertebroplasty group	Kyphoplasty group	Mean age (years)	Mean duration of symptoms (weeks)	Follow-up duration (months)	Outcome measures	Conclusion
Liu et al. [45]	2010	Taiwan	100	50	50	73.3	2.3	6	Visual analog scale	Small differences between the two groups
Dohm et al. [46]	2014	USA	381	190	191	75.6	12	24	SF-36 physical component summary, EQ-5D quality-of-life, Oswestry disability index	Similar clinical improvement
										Vertebroplasty: Shorter procedure duration and hospitalization
										Kyphoplasty: fewer cement leakages, longer fracture-free survival, less loss of kyphotic-deformity correction
Evans et al. [47]	2016	USA	115	56	59	75.6	9.4	12	Pain (0–10 scale), Roland-Morris Disability Questionnaire	Equally effective

### Economic cost consideration of treatment methods

The cost-effectiveness of vertebral augmentation has been thoroughly analyzed. Studies carried out in the UK [50] and the US [51] demonstrated that vertebral augmentation is initially more expensive than non-operative management, however, it is cost-effective as it improves survival rates. A recent systematic review by Pron et al. [52] also demonstrated that vertebral augmentation leads to shorter hospital stays and higher early health gains. Non-operative treatment has also been associated with higher post-acute care costs than vertebral augmentation in some studies [53, 54]. These findings raise the consideration that patients who have multiple risk factors for failure of non-operative management may benefit from early surgical intervention.

### Prospects of operative management

In recent years, new methods of augmentation have been developed to address the cement leakage and the potential side effects associated with it. Radiofrequency-targeted vertebral augmentation is a different type of kyphoplasty; ultra-high-viscosity cement is injected into channels created in the body of a vertebra using radiofrequency. Cement is infused at a much slower and more controlled rate to minimize leakage [55]. As a result, this modified procedure may provide up to 50% reduction in the number of adverse effects due to cement leakage when compared with standard kyphoplasty [56].

To decrease cement leakage, other techniques have been developed for percutaneous vertebral augmentation, such as the injection of cement in devices used to maintain height correction after deflation of a kyphoplasty balloon. These devices include a porous vessel balloon, titanium [57], and coil-filled systems [58]. Another device that has been introduced is augmentation with titanium expandable supports [59]. Expandable vertebral devices theoretically increase stiffness and decrease cement leakage and adjacent-level fractures. Nevertheless, these techniques need the support of high-quality studies to test their efficacy and cost-effectiveness.

## Secondary fracture prevention

Secondary fracture prevention is an essential part of fragility fracture management. It has been proven that patients who suffer a first fragility fracture have an 86% increase in the probability of suffering a second fracture if they are left without the appropriate antiosteoporotic treatment [60, 61]. The presence of a vertebral fracture, even if it is asymptomatic, increases the risk of new vertebral fractures by four- to fivefold [62]. The vertebral body immediately adjacent to a fractured vertebra is at a higher risk of suffering an osteoporotic fracture and causing a positive feedback cycle termed “vertebral fracture cascade” [63, 64]. Additionally, OVFs are often precursors of hip fractures, and therefore, appropriate antiosteoporotic treatment is necessary for their prevention [65, 66].

Although the consequences of osteoporosis underdiagnosis and undertreatment are well-documented, screening and treatment rates remain low [67]. Barton et al., reported that from 2933 patients presented with a vertebral fracture to the emergency department of a level I trauma center or its affiliated hospitals, only 2% received a DXA scan and 5% started antiosteoporotic treatment within one-year post-fracture, and subsequently 38% of them suffered a second osteoporotic fracture within 2 years [67].

Antiosteoporotic medication can vary. A recent meta-analysis by Jin et al. reported that zoledronate, alendronate, risedronate, etidronate, ibandronate, minodronate, pamidronate, PTH, denosumab, romosozumab, and SERMs had significant secondary prevention effects on OVFs [68]. However, zoledronate, risedronate, and PTH demonstrated noteworthy effects in preventing non-vertebral fractures in patients with vertebral fractures. A recent database study demonstrated that initiation of antiosteoporotic medication is the most important step to stop the vertebral fracture cascade, as it has been associated with a 19% decreased risk of secondary vertebral fractures [69]. On the other hand, surgical treatment with vertebral augmentation techniques does not alter secondary fracture risk compared to non-operative management [69]. As a result, appropriate medical treatment may preserve spine biomechanics more effectively than vertebral augmentation.

Osteoporosis screening and initiation of treatment during the hospitalization of the patient is an effective method of enhancing secondary fracture prevention [70, 71]. Nevertheless, it should be kept in mind that many patients with fragility fractures are not admitted to the hospital [72]. Surveys have shown that the absence of an established care pathway for fragility fracture patients creates miscommunication between the patient and the different medical specialties [73]. The Fracture Liaison Service (FLS) is a proven way to close the secondary fracture prevention care gap and eliminate this confusion. An FLS unit consists of a multidisciplinary team of healthcare professionals, with the objectives of identifying patients with fragility fractures, assessing osteoporosis, initiating appropriate treatment, and improving long-term adherence to the treatment [74]. In patients with OVFs, the effectiveness of FLS in reducing secondary fracture rates has been well-documented [74].

Despite its proven value, the FLS implementation is limited due to the financial restrictions of healthcare systems [75]. Several organizations, such as the Fragility Fracture Network (FFN) and the International Osteoporosis Foundation (IOF), have made significant efforts to expand FLS implementation [76]. A recent meta-analysis by Danazumi et al., included 37 studies that investigated the effects of FLS interventions compared to non-FLS interventions and demonstrated that FLS patients had a 32% decreased secondary fracture risk at 2 years of follow-up [77].

## Summary

OVFs are the most common osteoporotic fractures and can be a cause of significant pain, loss of function, and mortality. Early diagnosis is essential to initiate appropriate treatment, and consequently, a high level of suspicion is required. In neurologically intact patients, the first line of treatment should be non-operative. Non-operative management entails analgesia, orthoses, and early mobilization. In patients with persisting pain despite non-operative care, surgical management with vertebral augmentation with percutaneous vertebroplasty or kyphoplasty can be offered. Secondary fracture prevention is essential, as refracture rates are high in patients who do not receive appropriate antiosteoporotic medication. Inpatient administration of antiosteoporotic medication has been shown to significantly increase persistence to medication. FLS is an effective model that facilitates follow-up of fracture patients and improves adherence to antiosteoporotic medication, therefore enhancing secondary fracture prevention, and as such, it should be more widely embraced by the healthcare systems.

## Data Availability

No datasets were generated or analyzed during the current study.
